# Relevance of MRI in prediction of malignancy of musculoskeletal system-A prospective evaluation

**DOI:** 10.1186/1471-2474-10-125

**Published:** 2009-10-08

**Authors:** Alex Daniel, Ekram Ullah, Shagufta Wahab, Vasantha Kumar

**Affiliations:** 1 Department of Radiodiagnosis, JNMCH, AMU Aligarh, India

## Abstract

**Background:**

The purpose of this study is to evaluate the role of MRI in musculoskeletal tumours, especially, in prediction of malignancy & to compare whether the diagnosis made on MRI correlates with the cytological/histopathological diagnosis.

**Methods:**

50 consecutive patients presenting in the Outpatient and Inpatient department of the Department of Orthopaedics or Casualty of Jawaharlal Nehru Medical College and Hospital, AMU, Aligarh, India were included in this study. They were subjected to MR examination on 1.5 Tesla superconducting system (MAGNETOM Avanto, Siemens). After localizer sequences, T1W and STIR images were obtained in longitudinal planes followed by T2W and post contrast T1W images in axial planes. Additional sequences were taken when required. Various imaging characteristics of tumours were evaluated statistically and their respective sensitivity and specificity in prediction of malignancy were obtained.

**Results:**

Features associated with benign diagnosis in a large percentage of cases, are size less than 8 cm, sharp margination, homogeneous T2 signal, absence of oedema, necrosis, calcification and fluid-fluid levels. Similarly, malignant tumours are commonly associated with presence of irregular margins, inhomogeneous signal intensity, oedema, necrosis, haemorrhage, fascial penetration, bone changes and neurovascular involvement. A correct histological diagnosis is reached on the basis of imaging studies alone in 65% to 75% of cases. The sensitivity for a MRI diagnosis of malignant tumour was 95% and specificity was 84%.

**Conclusion:**

Differentiation of malignant from benign lesions of musculoskeletal system is best made by a combination of clinical and imaging parameters rather than by any single MR characteristic. When a lesion has a non-specific MR imaging appearance, it is useful to formulate a suitably ordered differential diagnosis based on tumour prevalence, patient age, and anatomic location. A systematic approach markedly improves diagnostic results.

## Background

The determination of the anatomical extent, characteristics, and histopathological features of bone tumors and soft-tissue sarcomas involves a diagnostic strategy in which a biopsy is the final step [1]. MRI, however, is usually the best imaging system for the evaluation of a soft-tissue mass or the extent of soft-tissue or bone-marrow involvement by a bone tumor [2]. MRI demonstrates the depth, size, and local extent of tumours. Published opinions regarding the value of MR imaging in characterizing the pathologic nature of musculoskeletal masses and discriminating between benign and malignant lesions are divergent. There is a wide range of specificity values of MR imaging in differentiation of benign from malignant musculoskeletal lesions reported in the literature. Berquist et al. in 1990 [3] and Moulton et al. in 1995 [4] found a relatively high specificity of 76%-90%. Other researchers have reported that MR imaging has low specificity in differentiation between benign and malignant masses, and most lesions demonstrate a nonspecific appearance [5]. Thus, the role of MRI in predicting malignancy has been inadequately studied in literature and here we venture to find out suitable imaging characteristics or a combination of them for prediction of malignancy and to compare whether the diagnosis made on MRI correlates with the histopathological/cytological diagnosis.

## Methods

The study included 50 patients that came from the department of Orthopaedics or Casualty during the period from October 2006 to September 2008, presenting with pain, a localized swelling in the limb or inability to use the limb, which seemed to be arising from the subcutaneous/muscle plane or underlying bone on preliminary clinical examination. Patients with superficial lesions, in which a definite diagnosis was possible on clinical grounds e.g. abscess were excluded from the study. The study was approved by the ethical committee of the hospital and written and informed consent was taken from the patients/attendants. Following detailed physical examination (including local examination of the swelling, vitals, systemic examination with emphasis on musculoskeletal system evaluation); they were subjected to MR examination on 1.5 T superconducting system (MAGNETOM Avanto, Siemens). T1W & STIR images were obtained in a sagittal or coronal plane, for accurate determination of longitudinal extent of tumour. Later, T2W & post-contrast T1W images were obtained in axial planes. Other sequences like GRE were obtained, as and when required. Diagnoses were confirmed with histopathological examination. Various MR imaging characteristics of benign and malignant musculoskeletal tumours were identified and they were evaluated prospectively for their role in prediction of malignancy. These characterstics included size of tumour, shape and lobulation, margination, signal intensity on T1 and T2 weighted sequences, enhancement pattern, homogenous or heterogenous appearance, peritumoral edema, presence of necrosis and calcification, fluid-fluid level, neurovascular involvement etc.

## Results

**Figure 1:**
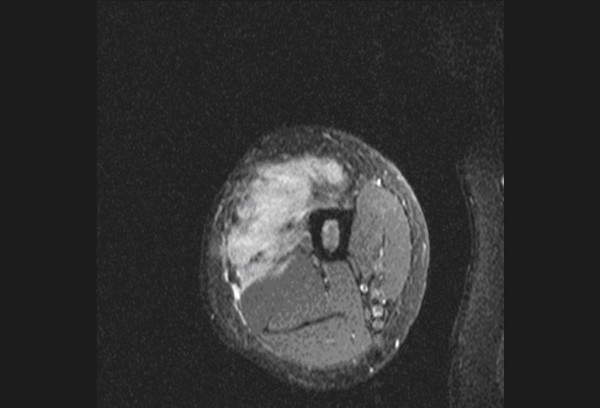
**Post-contrast T1 fat saturated (FS) axial image of upper thigh shows irregular infiltrative margins of the malignant fibrous histiocytoma**.

**Figure 2:**
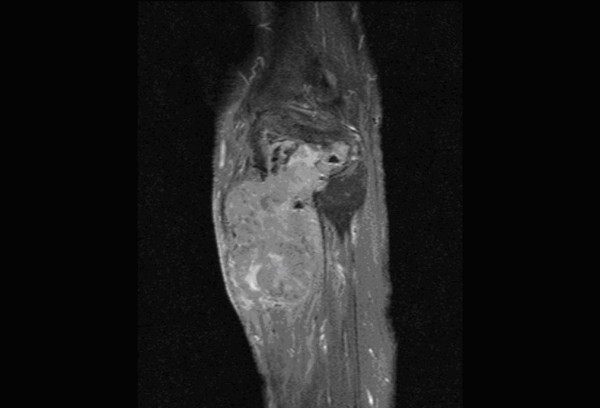
**Post-contrast T1 FS sagittal image of upper leg shows heterogeneous nature of the malignant giant cell tumour**.

**Figure 3:**
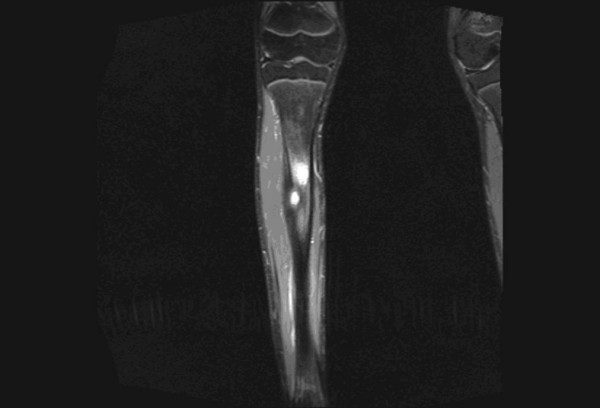
**STIR coronal image of leg shows gross peritumoral edema in a patient with osteoid osteoma of tibia**.

**Figure 4:**
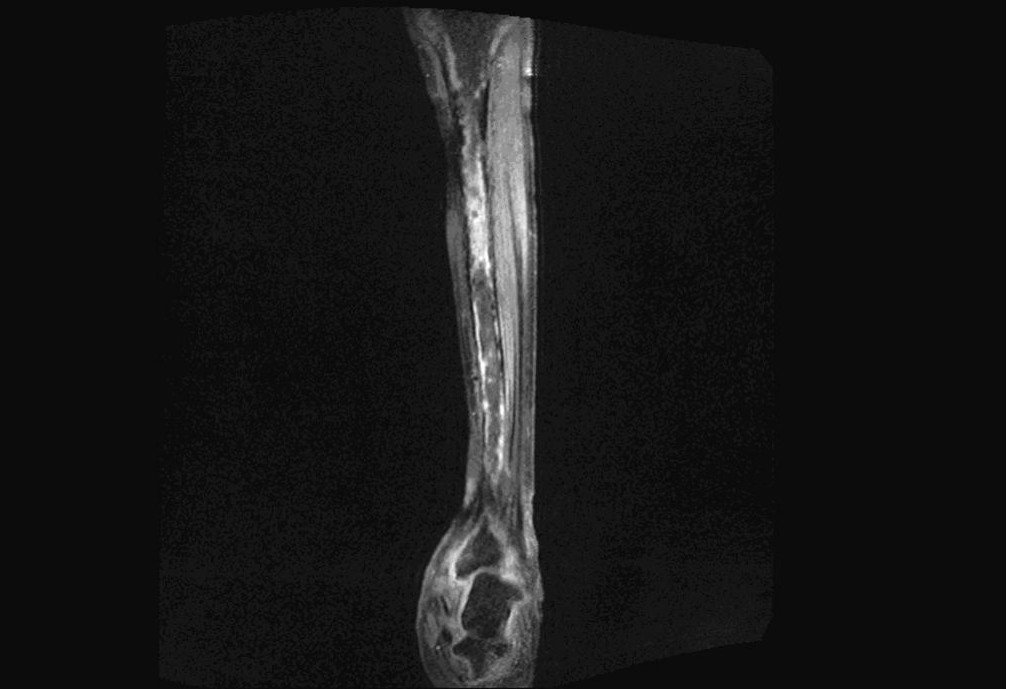
**Post-contrast T1 FS coronal image of leg shows evidence of intratumoral necrosis**.

**Figure 5:**
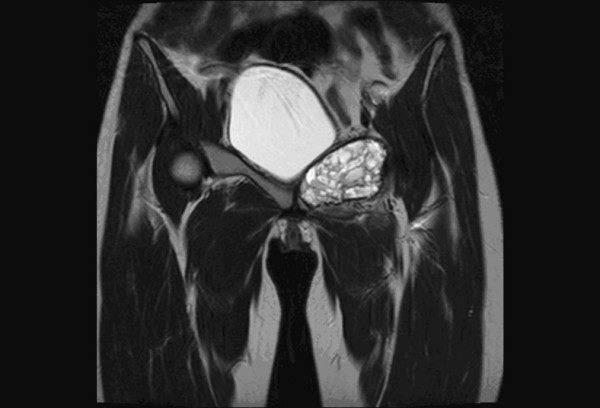
**Coronal T2WI image of pelvis shows fluid -fluid levels with evidence of hemorrhagic foci in a patient with aneurysmal bone cyst of ischiopubic ramii**.

**Figure 6:**
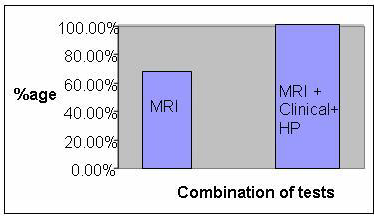
**Bar Diagram showing the various percentage of diagnosis reached with MRI and Combination of modalities**.

Out of the 50 patients included in the study, 32 (64%) were males and 18 (36%) were females. The age-range of patients included in this study was from 11 to 80 (Table 1). The proportion of benign and malignant tumours confirmed on histopathological evaluation was 52% (26) and 48% (24) respectively. Of these tumours seemed to be arising from bone in 32 (64%) and from soft tissue in 18 (36%) cases. Malignant tumours were seen in 19 out of 32 male patients (59%) and 5 out of 18 female patients (28%). The MR imaging characteristics included in this study with their respective sensitivity and specificity are tabulated in Table 2. A number of features were associated with a benign diagnosis, including size less than 8 cm, sharp margination, homogeneous T2 signal, absence of edema, and absence of necrosis or calcification and fluid-fluid levels (FFLs). Similarly, malignant tumours are commonly associated with size more than 8 cm, irregular margins (Figure 1), inhomogeneous signal (Figure 2), and presence of edema (Figure 3), necrosis (Figure 4), hemorrhage (Figure 5), fascial penetration, bone changes and neurovascular involvement. MRI diagnosis of the tumor as benign or malignant was made subjectively based on a combination of MRI features, tumor prevalence and location of tumor as well as patient's age and sex. Three senior radiologists made the diagnosis and in cases of contradiction, the diagnosis was made based on majority decision. A correct diagnosis is reached on the basis of imaging studies alone in 65% to 75% of cases (Figure 6). The MRI diagnosis & final diagnosis were compared and the results are tabulated in Table 3.

**Table 1:** Demographics of Subjects

**Age Group**	**Benign**	**Malignant**	**Grand Total**
11-20	8	11	19

21-30	7	5	12

31-40	4	3	7

41-50	4	3	7

51-60	1	2	3

71-80	2	0	2

Grand Total	26	24	50

**Table 2:** Statistical values of various MRI features in prediction of malignancy

**S.n**	**Criterion**	**Sensitivity**	**Specificity**	**PPV**	**NPV**
1.	Size > 6 cm	0.95	0.57	0.67	0.93

2.	Size >8 cm	0.75	0.76	0.75	0.76

3.	Shape: irregular, lobulated	0.83	0.76	0.76	0.83

4.	Margins: infiltrative	0.91	0.65	0.70	0.89

5.	Isointensity on T1W	0.70	0.76	0.73	0.74

6.	Slight hyperintensity on T2W	0.95	0.38	0.58	0.90

7.	heterogeneous lesion	1	0.5	0.64	1

8.	Peritumoral edema	0.95	0.5	0.63	0.92

9.	Absence of multiplicity	0.54	0.15	0.37	0.26

10.	Intratumoral necrosis	0.87	0.84	0.84	0.88

11.	Intratumoral hemorrhage	0.70	0.65	0.65	0.70

12.	Intratumoral calcification	0.70	0.88	0.85	0.76

13.	Absence of Intratumoral fat	1	0.07	0.5	1

14.	Absence of Intratumoral fibrosis	0.87	0.03	0.45	0.25

15.	Fascia penetration	0.83	0.73	0.82	0.74

16.	Bone changes	0.83	0.84	0.83	0.84

17.	Neurovascular involvement	0.83	0.88	0.86	0.85

18.	Enhancement pattern (Heterogenous)	1	0.07	0.5	1

19.	Absence of T1 hyperintense tracts	1	0.11	0.51	1

20.	Absence of Fluid-fluid levels	0.83	0.26	0.51	0.63

**Table 3:** Specific diagnoses made or suspected on the basis of MR imaging

**MRI Diagnosis**	**Final diagnosis**		
	
	**Benign**	**Malignant**	**Total**
Benign	22	1	23

Malignant	4	23	27

Grand Total	26	24	50

## Discussion

Overall prevalence of malignant musculoskeletal tumours is estimated between 5.1 and 15.5% of all sarcomas [6]. In our study the relatively high number (n = 24, 48%) of malignant lesions was due to a selection bias caused by the referral policy including only patients who had an MR examination, excluding a large number of (superficial) lesions treated without imaging and of typically benign "do not touch" lesions. The low overall prevalence rate of musculoskeletal tumours is probably due to the fact that the referring centres are requested to send all musculoskeletal tumours (benign and malignant) to the national registry.

In our study, among the morphological characteristics, size criteria of >6 cm and >8 cm yielded a sensitivity of 95% and 75% respectively. However, size criteria of >8 cm had a specificity of 76% while >6 cm had a specificity of 57%. Irregular and lobulated shapes of the tumors had a sensitivity and specificity of 83% and 76% respectively. Irregular and infiltrative margins (Figure. 1) had a sensitivity and specificity of 91% and 65% respectively. Berquist et al in 1990 [3] conducted a study on 95 consecutive patients with soft tissue mass lesions and observed that 87% of malignant tumours were larger than 5 cm. 85% of malignant tumours had irregular margins. Moulton et al in 1995 [4] showed that size criteria of >5 cm had a sensitivity of 85% and irregular margins had a sensitivity of 74%. So these morphologic characteristics have varied sensitivities in various studies and cannot be reliably used for differentiating benign from malignant tumours. Benign lesions tend to have well defined margins, and some benign masses have characteristic appearances that aid in their differentiation from malignant processes [7]. In our study, 'Heterogeneous appearance' of the tumour (Figure. 2) had a sensitivity and specificity of 100% and 50% respectively. 'Presence of peritumoral edema' (Figure. 3) had a sensitivity and specificity of 95% and 50% respectively. These characteristics are highly sensitive, but the specificity is too low to be considered reliable differentiating factors. De Schepper et al in 1992 [8] noted inhomogeneous signal in 88% of malignant tumours. He had reported highest sensitivity for "absence of low signal intensity on T2" (100%). Pang et al, in 2003 [9] demonstrated that statistically significant imaging features favouring a diagnosis of malignancy included inhomogeneity on T2-weighted images (p = 0.002) and a change in pattern from homogeneity on T1-weighted images to inhomogeneity on T2-weighted images (p = 0.003). Brüning et al, in 1993 [10] evaluated the incidence, quantity, and presentation of intra- and extraosseous edema accompanying benign and malignant primary bone lesions. The mere presence and quantity of marrow and soft tissue edema are unreliable indicators of the biologic potential of a lesion [10]. Golfieri et al in 1991 [11] studied the MR morphologic appearance of primary bone tumors correlated with pathologic examinations and observed that peritumoral soft tissue edema was found by STIR sequence only in malignant tumors. Crim et al. in 1992 [12] and Griffiths et al. in 1993 [13] also studied the morphologic characteristics of tumours and observed that the majority of both benign and malignant masses had inhomogeneous signal intensity and at least partially irregular borders and MR imaging can be used to evaluate the extent of soft-tissue masses, but most masses will require biopsy to determine if they are benign or malignant.

In our study, other imaging parameters like 'Intratumoral necrosis' (Figure. 4) had a sensitivity and specificity of 87% and 84% respectively. 'Intratumoral hemorrhage' (Figure. 5) had a sensitivity and specificity of 70% and 65% respectively. 'Intratumoral calcification' had a sensitivity and specificity of 70% and 88% respectively. The comparatively decreased specificity of 'Intratumoral hemorrhage' is due to large number of aneurysmal bone cysts in the study. Hemorrhage or edema or both were observed in 64% of malignant tumours [1]. Hemorrhage was noted in 57% of malignant tumours [4]. Alyas et al in 2007 [14] studied prevalence and diagnostic significance of fluid-fluid levels in soft-tissue neoplasms and found that the presence of FFLs does not reliably distinguish benign from malignant neoplasms, although all lesions with more than two-thirds FFLs were benign. Other imaging parameters which are directly related to the aggressive nature of the tumour like 'fascia penetration', 'bone erosion' and neurovascular involvement had high specificity and sensitivity in the order of 88% and 81% respectively. These values are comparable with the values derived in other prospective and retrospective studies conducted earlier [3,4]. The highest sensitivity and specificity combination was observed for neurovascular involvement, Intratumoral necrosis, bone erosion or invasion and fascia penetration. Enhancement pattern, particularly, heterogenous pattern of enhancement was very helpful in making the diagnosis. The maximum sensitivity was observed for 'heterogeneous appearance' of malignant tumours. The maximum specificity was observed for 'neurovascular involvement'. In our study, we had classified the tumours into benign and malignant based on imaging parameters described above. Then these diagnoses were correlated with histopathological diagnosis of a benign or malignant tumor. The MRI diagnosis & final diagnosis were compared (Please refer to Table 3). Retrospective studies on the differentiation of malignant from benign musculoskeletal tumours by MR imaging largely outnumber prospective ones. De Schepper et al. in 1992 [8] performed retrospectively a multivariate statistical analysis to determine the accuracy of ten parameters, individually and in combination, for predicting malignancy. A sensitivity and specificity of 81% was achieved when a combination of parameters was used.

Prospective studies by Ma et al., 1995, [15] Berquist et al., 1990 [3] and Moulton et al., 1995, [4] respectively, a sensitivity of 100, 94 and 78% and a specificity of 17, 90 and 89% for predicting malignancy were reported. The high sensitivity (100%) in the study of Ma et al. coincides with a very low specificity (17%) caused by a rigorous threshold of parameters that differentiates benign form malignant lesions, avoiding all false negatives. The additional value of MRI for differentiating benign from malignant lesions in these circumstances is doubtful. In most previous studies, the accuracy of MRI was evaluated by using a combination of quantitative parameters. In a retrospective study of 44 cases, however, Teo et al. in 2000, [16] concluded that malignant soft tissue masses are reliably distinguished from hemangioma by subjective analysis combining lesion morphology, signal intensity and enhancement after gadolinium chelate injection. The subjective method for the differentiation of malignant from benign musculoskeletal tumours is also supported by Berquist et al [3]. They could not identify any quantitative criterion or combination of criteria that could differentiate benign from malignant musculoskeletal tumours with greater accuracy than on subjective evaluation. In our study, analysis of different imaging characteristics was performed in order to obtain a specific diagnosis or differential diagnosis with a maximum of three possibilities but basically differentiating the possibility of benign and malignant lesion. The patient's age, sex and clinical presentation were also used. We used imaging characteristics as described in the methods section, but also considered tumor prevalence, location, age of the patient and concomitant diseases in establishing the diagnosis. Compared to the study of Moulton, [4] our study, showed that MRI reliably identifies malignancy in musculoskeletal tumours with a higher sensitivity (95.83 vs. 78%) with high negative predictive value (NPV) (95%) and comparable specificity (84 vs. 87%), but with rather high positive predictive value (PPV) (85%). Better sensitivity was probably the result of a methodology adapted to clinical radiological practice (three diagnostic possibilities in our study and only one MRI diagnosis in Moulton's study), progress in radiological science with inclusion of newly described parameters, description of larger series of tumors with specific imaging characteristics and the diagnostic skill of radiologists. A large prospective study showed that differentiating malignant from benign lesions in these tumours is best achieved by using a combination of clinical and imaging parameters rather than by any single MRI feature [4].

Gielen et al performed a prospective non-quantified MR parameter evaluation in patients with soft tissue tumors. It showed that differentiation between malignant and benign lesions (dignity), a sensitivity of 93%, specificity of 82%, negative predictive value (NPV) of 98% and positive predictive value (PPV) of 60% with accuracy of 85%. For benign lesions the sensitivity was 75% and for malignant lesion it was 37%. A correct diagnosis was proposed in 50% of the cases which was later confirmed on histopathology [17].

Rijswijk et al observed that the use of combined nonenhanced static and dynamic contrast-enhanced MR imaging demonstrated the finest diagnostic performance in the prediction of soft-tissue tumors [18]. Contrast-enhanced MR imaging parameters that favored malignancy were liquefaction, early dynamic enhancement (within 6 seconds after arterial enhancement), peripheral or inhomogeneous dynamic enhancement, and rapid initial dynamic enhancement followed by a plateau or washout phase [18]. They addressed the controversial issue of routine use of contrast agent in mucsuloskeletal tumors with the point that it, gadopentetate dimeglumine greatly improved not only the detection of benign lesion but also malignant lesions [18].

## Conclusion

No single characteristic consistently allowed distinction of benign from malignant tumors. Malignancy is predicted with the highest sensitivity when lesions have high signal intensity on T2-weighted images, larger than 6 cm diameter, have heterogeneous signal intensity on T1-weighted images and have peritumoral edema. The highest specificity is noted when lesions show tumor necrosis, bone or neurovascular involvement and mean diameter of more than 8 cm. When a lesion has a non-specific MR imaging appearance, it is useful to formulate a suitably ordered differential diagnosis based on tumour prevalence, patient age, and anatomic location. A systematic approach markedly improves diagnostic results.

## Competing interests

The authors declare that they have no competing interests.

## Authors' contributions

EU conceived of the study and the design. SW conceived, participated in design and coordinated the study. SW involved in correspondence as well. AD and VK were involved acquisition, analysis and interpretation of data. All the authors have read and approved the final manuscript.

## Pre-publication history

The pre-publication history for this paper can be accessed here:


